# Simultaneous overexpression of ∆6-, ∆12- and ∆9-desaturases enhanced the production of γ-linolenic acid in *Mucor circinelloides* WJ11

**DOI:** 10.3389/fmicb.2022.1078157

**Published:** 2022-12-15

**Authors:** Xiuwen Wang, Junhuan Yang, Hassan Mohamed, Aabid Manzoor Shah, Shaoqi Li, Shuxian Pang, Chen Wu, Futing Xue, Wenyue Shi, Beenish Sadaqat, Yuanda Song

**Affiliations:** ^1^Colin Ratledge Center for Microbial Lipids, School of Agricultural Engineering and Food Science, Shandong University of Technology, Zibo, China; ^2^Department of Food Sciences, College of Food Science and Engineering, Lingnan Normal University, Zhanjiang, China; ^3^Department of Botany and Microbiology, Faculty of Science, Al-Azhar University, Assiut, Egypt

**Keywords:** desaturases, co-overexpression, homologous recombination, *Mucor circinelloides*, GLA production

## Abstract

*Mucor circinelloides* WJ11, an oleaginous filamentous fungus, produces 36% lipid of its cell dry weight when cultured in a high C/N ratio medium, however, the yield of γ-linolenic acid (GLA) is insufficient to make it competitive with other plant sources. To increase the GLA content in *M. circinelloides* WJ11, this fungus was engineered by overexpression of its key genes such as Δ6-, Δ12-, and Δ9-desaturases involved in GLA production. Firstly, we tried to overexpress two Δ6-desaturase isozymes to determine which one played important role in GLA synthesis. Secondly, Δ6-and Δ12-desaturase were co-overexpressed to check whether linoleic acid (LA), the precursor for GLA synthesis, is a limiting factor or not. Moreover, we tried to explore the effects of simultaneous overexpression of Δ6-, Δ12-, and Δ9-desaturases on GLA production. Our results showed that overexpression (1 gene) of *DES61* promoted higher GLA content (21% of total fatty acids) while co-overexpressing (2 genes) *DES61* and *DES12* and simultaneous overexpressing (3 genes) *DES61*, *DES12*, and *DES91* increased the GLA production of engineered strains by 1.5 folds and 1.9 folds compared to the control strain, respectively. This study provided more insights into GLA biosynthesis in oleaginous fungi and laid a foundation for further increase in GLA production into fungus such as *M. circinelloides*.

## Introduction

In recent years, polyunsaturated fatty acids (PUFAs) have received great interest in the nutritional and pharmaceutical fields. PUFAs are structural components of cell membranes and regulate the fluidity and stability of membranes, as well as precursors of eicosanoids such as prostaglandins, leukotrienes, and thromboxanes (Certik et al., 1998; Ge et al., 2017). γ-Linolenic acid (GLA, 18:3, ω-6) and α-linolenic acid (ALA, 18:3, ω-3) are regarded as essential fatty acids (EFAs), however, mammals lack the ability to synthesize these EFAs (Cui et al., 2020). The *de novo* synthesis of omega-3 (ω-3) and omega-6 (ω-6) polyunsaturated fatty acids is derived from ALA and GLA respectively, and catalyzed by a complex series of desaturases and elongases consecutively (Pereira et al., 2003). Δ6-desaturase catalyzes the synthesis of GLA from linoleic acid (LA), which makes it a key regulatory step for PUFA formation (Certik et al., 1998; Jiang et al., 2017). GLA plays a crucial role in the prevention and treatment of diabetes, atopic dermatitis, cardiovascular diseases, inflammatory diseases, cancer, and other related diseases (Fan and Chapkin, 1998).

**Figure 1:**
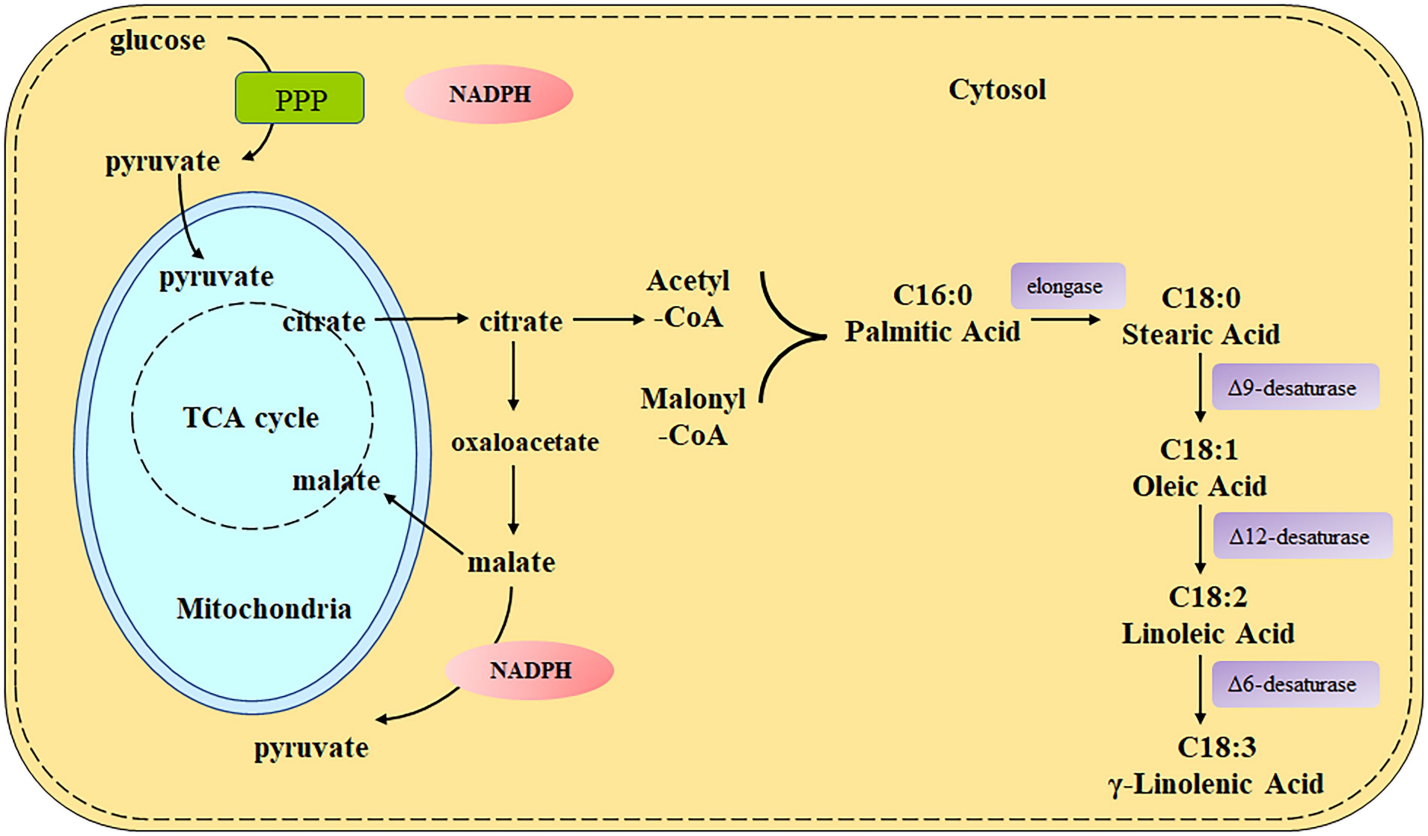
The metabolic pathway of GLA synthesis in *Mucor circinelloides*.

Nevertheless, the predominant conventional sources of the GLA-rich oil are a limited number of plant seeds, such as evening primrose (*Oenothera biennis*), borage (*Borago officinalis*), and blackcurrant (*Ribes nigrum*), which are vulnerable to season, climate, and arable land and cannot meet the increasing commercial demand (Čertík et al., 2012). Certain oleaginous fungi, such as *Mucor* spp., *Mortierella* spp., *Cunninghamella* spp., *Rhizopus* spp., and *Trichoderma* spp., are promising alternative microbial sources of GLA (Certik and Shimizu, 1999; Čertík et al., 2012). Moreover, *Mucor circinelloides* is the first strain that was used for GLA production by large-scale fermentation since the 1980s (Ratledge and Wynn, 2002), and has been used as a model organism for the study of fatty acid biosynthesis due to its available genetic information and sophisticated tools for genetic manipulation. *M. circinelloides* WJ11 has been isolated and considered a high lipid producer as it can accumulate lipid content up to 36% of its cell dry weight (CDW) with about 13% of GLA content (Tang et al., 2015a), which can be further increased by genetic manipulation and metabolic engineering (Yang et al., 2021; Zhang et al., 2021). The biosynthesis pathway of GLA in *M. circinelloides* has been elucidated clearly as shown in Figure 1. Starting from stearic acid (SA, C18:0), Δ9-desaturase leads to the production of oleic acid (OA, C18:1), which is further desaturated by Δ12-desaturase to linoleic acid (LA, C18:2). Finally, LA is converted to γ-linolenic acid (GLA, C18:3) by Δ6-desaturase, which acts as a precursor for other PUFAs synthesis (Zhang et al., 2017).

So far, microbial-derived GLA has not reached the productivity required for commercial production yet. Therefore, overexpression of key enzymes in the GLA biosynthesis pathway were used to increase GLA production. In recent years, with the emergence of advanced genetic manipulation techniques, the isolation, identification, and homologous or heterologous expression of desaturases and elongases have become the focus of active research (Wang et al., 2007; Chuang et al., 2010; Wu et al., 2021). There is a great interest in improving the production of GLA through overexpression of some key desaturase genes involved in the GLA biosynthesis pathway. The ∆6-desaturase genes from *Cunninghamella echinulate* and *Rhizopus stolonifer* were cloned and overexpressed in *Komagataella phaffii*, and the GLA contents were up to about 3.1 and 22.4%, respectively (Wan et al., 2009, 2011). Heterologous expression of ∆6-desaturase from *Mucor rouxii* in *Saccharomyces cerevisiae* provided with exogenous LA, produced 7.1% GLA of total fatty acids in the mutant (Laoteng et al., 2000). Various studies have reported the expression of ∆12-desaturase which introduces a double bond into OA to generate LA that serves as a precursor substrate for GLA synthesis (Passorn et al., 1999; Wang et al., 2016; Lamers et al., 2019). In *S. cerevisiae*, the heterologous overexpression of ∆12-desaturase resulted in the LA content up to 25% of the total fatty acids, and heterologous overexpression of ∆6-desaturase resulted in the GLA content up to 10% (when supplemented with exogenous LA), meanwhile, the co-overexpression of ∆6- and ∆12-desaturases resulted in the GLA content up to 8% (Huang et al., 1999). Chuang et al. achieved the generation of GLA (up to 20%) by heterologous co-overexpressing ∆6- and ∆12-desaturase genes from *Mortierella alpina* in *Yarrowia lipolytica* (Chuang et al., 2010). Because of the substrate specificity, expression of heterologous genes may lead to inefficiency. However, we previously investigated the role of homologous overexpression of ∆61, ∆62-desaturase, and co-overexpression of ∆61- and ∆12-desaturase in *M. lusitanicus* CBS277.49, formerly classified as *M. circinelloides f. lusitanicus* (Wagner et al., 2020). In that study, the single overexpressing ∆61-desaturase strain accumulated GLA yields up to 180 mg/l which is equal to a 38% increase in GLA content (from 31.2 to 43%) more than that of the ∆6- and ∆12-desaturase co-overexpression strain (Zhang et al., 2017). Laoteng et al. cloned and identified a ∆9-desaturase gene from *Mucor rouxii* that catalyzes the synthesis of monounsaturated fatty acids as substrates for GLA synthesis (Laoteng et al., 1999; Lee et al., 2021). In another study done by Qiao et al. the lipid content was increased by 25% *via* overexpressing ∆9-desaturase in *Y. lipolytica* (co-overexpressed by ACC1 and DGA1) (Qiao et al., 2015). However, the role of the simultaneous overexpression of the ∆9-, ∆12-, and ∆6-desaturase genes in GLA accumulation and lipid content is still unclear.

In this work, we investigated *M. circinelloides* WJ11, which accumulated higher lipid content (36% of CDW) than CBS277.49 (only 15% of CDW) (Tang et al., 2015b). The genomic analysis showed that there are two ∆6-desaturase isozymes (designated as ∆61- and ∆62-desaturase), one ∆12-desaturase, and two ∆9-desaturase isozymes (designated as ∆91- and ∆92-desaturase) coding genes in *M. circinelloides* WJ11. To investigate the effect of overexpression of different desaturase genes on GLA production in WJ11, we constructed the different engineered strains by inserting different combinations of *DES61*, *DES62*, *DES12*, *DES91*, and *DES92* genes into the genome of WJ11 by homologous recombination. The growth characteristics, expression levels of key genes, lipid accumulation, and fatty acid composition of transformants were analyzed. This is the first time to explore the role of simultaneous overexpression of desaturases in the GLA production of *M. circinelloides* WJ11.

## Materials and methods

### Strains, cultivation, and transformation conditions

The *Escherichia coli* Top 10 (vector for cloning and plasmid storage) was cultivated in Luria-Bertani (LB) media (Hanahan, 1983), at 37°C, shaking at 200 rpm. The wild-type *M. circinelloides* WJ11 (CCTCC no. M2014424) was used as a cDNA library of *DES61*, *DES62*, *DES12*, *DES91*, and *DES92*. The uracil and leucine auxotrophic strain MU760 of *M. circinelloides* WJ11 (host strain for target genes) was maintained in the complete YPG media supplemented with 200 μg/ml uracil or 20 μg/ml leucine when required (Bartnicki-García and Nickerson, 1962). The temperature was maintained at 28°C, and the pH was adjusted to 4.5 or 3.2 for the growth of mycelia or colonies, respectively. The transformation was conducted as previously described (Garre et al., 2015), linearized fragments were electroporated into fungal protoplasts and the transformants were selected using YNB media (Lasker and Borgia, 1980) or MMC media (Nicolas et al., 2007). The K & R medium (Kendrick and Ratledge, 1992b) was used as seed medium. Every recombinant strain (1 × 10^7^ spores) was inoculated in 100 ml K & R medium in 1 l baffled flasks, shaking at 130 rpm for 24 h, and then employed at 10% (v/v) to a 1.5-L fermenter with 1 l modified K & R medium which contained 80 g/l glucose and 2 g/l diammonium tartrate. The fermentation processes were carried out in small fermenters, stirred at 600 rpm, with 1.0 VVM aeration and pH controlled at 6.0 by 2 M NaOH for 4 days.

### Plasmid construction

The gene sequences of Δ6-desaturases (*DES61* and *DES62*), Δ12-desaturase (*DES12*), and Δ9-desaturases (*DES91* and *DES92*) were PCR-amplified from the WJ11 total cDNA with corresponding primers (Supplementary Table 1). The amplified PCR fragments, *DES61* and *DES62*, were cloned into vector pMAT2075 (Yang et al., 2020) digested by *XhoI* restriction endonuclease to construct DES61-overexpressing and DES62-overexpressing plasmids pCRC124 and pCRC125, respectively (Supplementary Figure 2). The plasmid pMAT2075 contained *pyrF* as a selection maker and strong promoter *pzrt1*, flanked by up- and down-stream 1 kb of *CarRP* sequences which can allow the replacement of carotenogenic *CarRP* gene (scaffold0226.18) on the WJ11 genome.

Another plasmid pMAT2076, which carried *LeuA* (Roncero et al., 1989) as a selection maker, also under the control of *pzrt1* and surrounded by up- and down-stream 1 kb of *sodit-a* sequences, was constructed as Supplementary Figure 1. The gene *sodit-a* (scaffold0239.15), encoding the plasma membrane malate transporter, was PCR-amplified from the WJ11 total DNA and its deletion has been shown to increase the lipid content (Yang et al., 2021). The primers F1/R1 were listed in Supplementary Table 1 and the *sodit-a* fragment with up- and down-stream sequences were digested with *SphI* and *SnaBI*. The primers F2/R2 (Supplementary Table 1) with *XbaI* and *NheI* digested sites were used to amplify linear pUC18 with up- and downstream sequences. Then the fragment was ligated with pUC18 (also digested with *SphI* and *SnaBI*) to obtain pUC18-sodita. The joined fragment *LeuA* and *pzrt1* was also digested with *NheI* and *XbaI*, and then the two fragments were ligated together to obtain pMAT2076. The *DES12* fragment was inserted into pMAT2076 to generate plasmid pCRC129 (Supplementary Figure 2). The plasmids pCRC150 and pCRC151 (Supplementary Figure 2), also derived from pMAT2076, were used for co-expression of *DES12* and *DES91*/*DES92* linked by a terminator T1 (Zhang et al., 2022) and another strong promoter *pgpd1* (Wolff and Arnau, 2002).

### Determination of cell dry weight, NADPH/NADP+ ratio, glucose, and ammonium ion concentration

Cultures of different engineered strains were collected at 3, 6, 12, 24, 36, 48, 60, 72, 84, and 96 h for biochemical analysis of the fermentation process. The mycelia were harvested by filtration through a Buchner funnel, washed with distilled water, and then kept at −80°C overnight. Samples were freeze-dried for 48 h to constant and the cell dry weight was measured gravimetrically. The values of NADPH/NADP^+^ were measured at 570 nm on a UV spectrophotometer according to the instructions of coenzyme II NADP(H) content assay kit (Nanjing Jiancheng Bioengineering Institute) (Yang et al., 2021). The concentration of glucose was determined by a glucose oxidase electrode biosensor (SBA-40E, Institute of Biology, Shandong Academy of Sciences, China) as described (Sun et al., 2017). The ammonium ion concentration was measured by indophenol method as described previously (Chaney and Marbach, 1962).

### Analysis of lipid content and fatty acids profile

Cellular lipids were extracted from fungal biomass according to the following procedures. Approximately, frozen 10 mg from each fungal biomass was vigorously homogenized using 2 ml of 4 M HCl, incubated at 80°C for 3 h. A 0.2018 g/100 ml of pentadecanoic acid (15:0 from Millipore, Sigma-Aldrich, United States) was added into the freeze-dried cell as an internal standard before methylation, methanolic HCl with 10% (v/v) was used for methylation at 60°C for 3 h. As Folch et al. described, chloroform/methanol (2:1, v/v) was added and the mixture was vortexed at room temperature for 15 min (Folch et al., 1956). Subsequently, 2 ml hexane was added to separate fatty acid methyl esters (FAMEs) for gas chromatography (GC) equipped with a 30 m × 0.32 mm DB-Waxetr column and a flame ionization detector. Nitrogen was used as carrier gas and the program was set at 120°C for 3 min, increasing at the speed of 5°C/min to 220°C for 2 min. The obtained chromatographic peaks and their retention times were determined by comparison to fatty acid methyl ester (FAME) standard mixture (Supelco 37-Component FAME Mix, Sigma-Aldrich, MO, USA). The lipid content was calculated by following formula:


$$\text{lipid content}=\frac{\frac{A_{1}}{A_{2}}\times c\times V}{m}\times 100\%$$

A_1_ = Total chromatographic peak area, A_2_ = Chromatographic peak area of internal standard, c = The concentration of internal standard, V=Volume of internal standard, m = Weight of fungal biomass. The lipid yield was calculated by lipid content multiplied by total biomass in grams per liter of fermentation sample. The GLA yield was calculated by percentage of GLA (quantified by GC) multiplied by total lipid content extracted from biomass.

### Molecular manipulation and analysis

Both wild type and mutants were grown for 4 days in 1 l K & R medium and fresh mycelia were collected for DNA extraction with the DNA Quick Plant System kit (Tiangen Biotech Co., Ltd). The fermentation samples harvested at 24 h were quickly frozen by liquid nitrogen and then ground fully using Trizol to obtain total RNA. RNA was reverse transcribed to cDNA using ReverTra Ace qPCR RT Kit (Roche) as described, previously (Wang et al., 2021). The Real-Time PCR (qPCR) was performed in LightCycle 96 (Roche) using the SYBR Green Realtime PCR Master Mix (Takara) referring to the instructions of the manufacturer, based on the 2^−ΔΔCt^ method. Primers used for RT-qPCR are shown in Supplementary Table 1.

### Statistical analysis

The statistical analyses were conducted *via* SPSS 21.0. All experiments were carried out in three independent replicates. Results were presented as the mean ± SD. *Student’s t* test was used to evaluate the differences between the means and *p* < 0.05 was considered as significantly different.

## Results

### Generation of *Mucor circinelloides* mutants overexpressing desaturase genes

**Figure 2:**
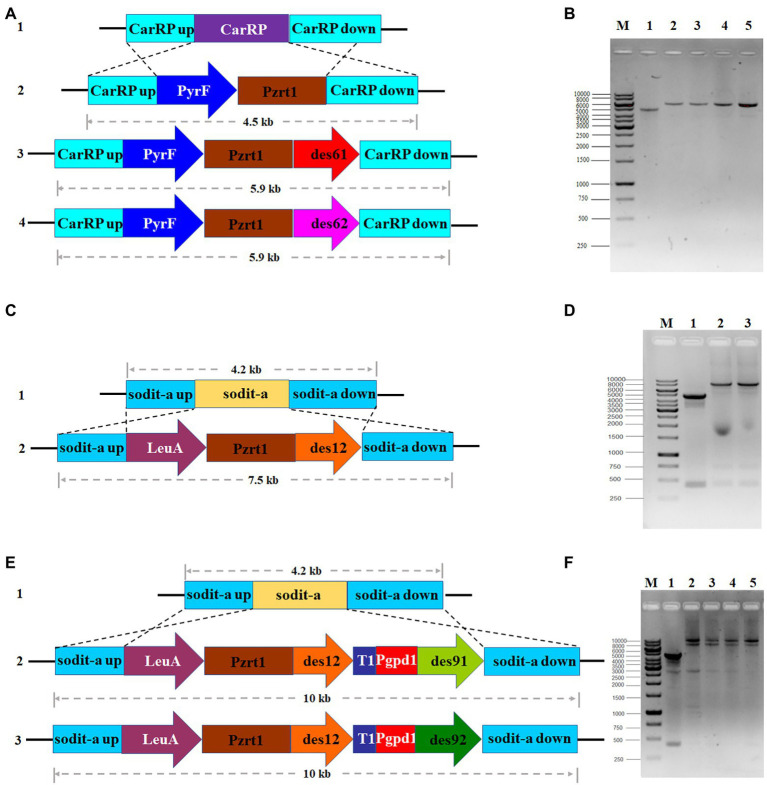
Overexpression of desaturase genes in *M. circinelloides* WJ11. **(A)** Genomic structure of MU760 (line 1), Mc-2075 (line 2), Mc-d61 (line 3) and Mc-d62 (line 4), **(B)** PCR amplification of control strains, Mc-2075 (lane 1), two transformants of Δ61-desaturase overexpressing strains (lane 2,3) and two transformants of Δ62-desaturase overexpressing strains (lane 4,5), **(C)** Genomic structure of Mc-d61 (line 1) and Mc-d61d12 (line 2), **(D)** PCR verification of Mc-d61 (lane1), two transformants of Δ61- and Δ12-desaturase co-overexpressing strains (lane 2,3), **(E)** Genomic structure of Mc-d61 (line 1), Mc-d61d12d91 (line 2) and Mc-d61d12d92 (line 3), **(F)** PCR amplification of Mc-d61 (lane 1), two transformants of Δ61-, Δ12- and Δ91-desaturase co-overexpressing strains (lane 2,3) and two transformants of Δ61-, Δ12-, and Δ92-desaturases co-overexpressing strains (lane 4,5).

The desaturase genes involved in the GLA production were identified by analyzing *M. circinelloides* WJ11 genomic data and alignment analysis from the National Center for Biotechnology Information (NCBI). They were named as *DES61* (encoded by scaffold00003.2), *DES62* (encoded by scaffold00010.52), *DES12* (encoded by scaffold00248.11), *DES91* (encoded by scaffold0005.3), and *DES92* (encoded by scaffold00126.19) (Tang et al., 2015b). Firstly, in order to investigate the role of two Δ6-desaturase isozymes in GLA production, the Δ61- and Δ62-desaturase overexpressing strains were generated. The plasmid pMAT2075, which is flanked by *carRP* sequences for homologous recombination, was used to receive target genes, *des61* and *des62*. Then the linear fragments from empty plasmid pMAT2075 (carried *pyrF* as selection maker) and target gene overexpressing plasmids pCRC124 (carried *des61*) and pCRC125 (carried *des62*) were transformed into the defective strain MU760 (uracil and leucine auxotrophic) to construct the strains Mc-2075, Mc-d61, and Mc-d62, respectively. Transformants were then screened as previously described (Rodriguez-Frometa et al., 2013) and verified by PCR with a pair of primers, carRP-F/R (Supplementary Table 1). The Mc-2075 was used as a control strain and two transformants for each mutant were selected for phenotype analysis. As shown in Figures 2A,B, the PCR products for each transformant were 4,476 bp (Mc-2075), 5,887 bp (Mc-d61-1 and Mc-d61-2), and 5,902 bp (Mc-d62-1 and Mc-d62-2), which were in accordance with the expected fragment sizes.

Secondly, to generate the Δ6-and Δ12-desaturase co-overexpression strains, two plasmids containing *DES6* and *DES12* were generated. The Δ6-desaturase overexpressing strains were constructed and Mc-d61 was selected as a candidate engineered strain for GLA production. Furthermore, Δ12-desaturase overexpressing plasmid was transformed into Mc-d61 strain to obtain Mc-d61d12 strain. The positive transformants were confirmed by a pair of primers, sodita-F/R (Supplementary Table 1). As shown in Figures 2C,D, the PCR fragments for each transformant were 4,217 bp (Mc-d61 and Mc-d62) and 7,510 bp (Mc-d61d12–1 and Mc-d61d12–2), in accordance with the expected fragment sizes.

Finally, given that Δ61-desaturase overexpressing plasmid was constructed, two gene expression cassettes (*DES12* and *DES91/DES92*) were inserted into one plasmid under different promoters *pzrt1* and *pgpd1* and then transformed into Mc-d61 to generate the Δ6-, Δ12-and Δ9-desaturase simultaneous overexpression strains, Mc-d61d12d91 and Mc-d61d12d92. The transformants were selected and verified by PCR with sodita-F/R (Supplementary Table 1) and the PCR products were found to be 4,217 bp (Mc-d61), 9,980 bp (Mc-d61d12d91–1 and Mc-d61d12d91–2) and 9,953 bp (Mc-d61d12d92-1and Mc-d61d12d92–2) as shown in Figures 2E,F, which were in accordance with the corresponding fragment sizes.

### The effect of two endogenous Δ6 desaturase isozymes on GLA production

**Figure 3:**
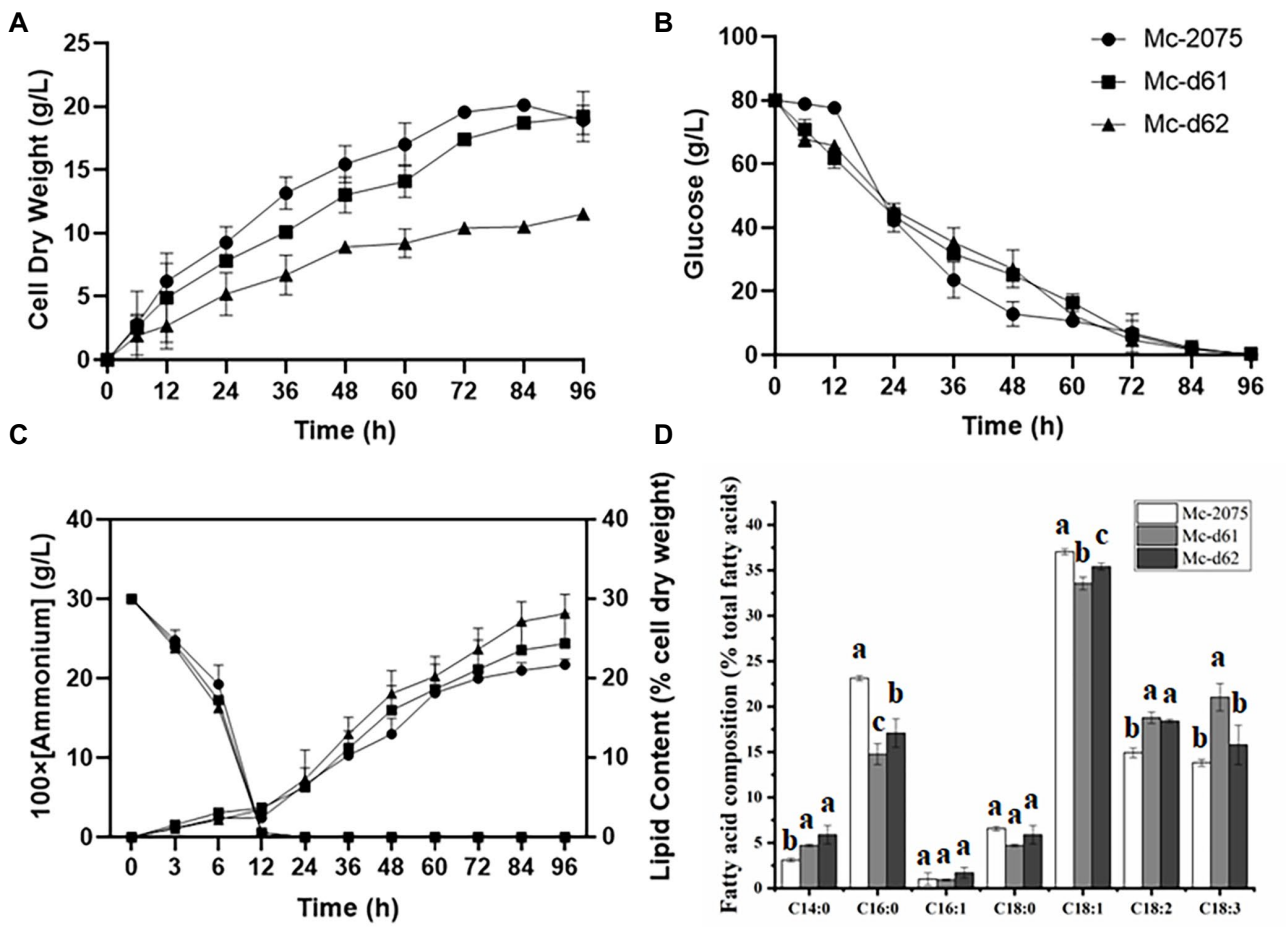
Fermentation parameters of the engineered strains with *DES61* and *DES62* overexpression. **(A)** Cell dry weight (CDW), **(B)** Glucose concentration, **(C)** Ammonium concentration and lipid content, **(D)** Fatty acid composition after 72 h fermentation in Mc-2075 (circle and white columns), Mc-d61 (square and gray columns) and in Mc-d62 (triangle and black columns) were measured when cultured in 1 l modified K & R medium for 96 h. The values were mean of three independent replicated experiments. Error bars represent the standard error of mean. Values with different letters were significantly different to each other.

As there was little difference in lipid content among the three transformants of each DES61/DES62-overexpression strain (data not shown), only one transformant was selected for subsequent analysis. Furthermore, due to the single overexpression strain being a leucine deficient strain, 20 μg/ml leucine was added into the medium to reduce the effects of *LeuA* deletion (Rodriguez-Frometa et al., 2013). In the medium with a high C/N ratio, the nitrogen source was depleted at 12 h during fermentation, and the lipids began to accumulate rapidly, reaching the peak at 24 h (Figure 3C). The carbon source (80 g/l of glucose) was exhausted within 96 h of fermentation (Figure 3B), and thereafter the biomass and lipid content increased slowly (after 72 h), what is more, lipids would be degraded after the carbon source was completely depleted. The cell dry weight of Mc-d61 and Mc-d62 was lower than that of the control strain throughout the whole fermentation process (Figure 3A). The growth pattern did not significantly change and the consumption level of glucose in medium was similar in all engineered strains (Figure 3B). The Δ61- and Δ62-overexpressing strains could accumulate more lipid than the control strain. The lipid content of Mc-d61 and Mc-d62 was up to 24.7 and 27.6% of cell dry weight, respectively, compared to the 22% of the control. In Mc-d61 and Mc-d62, the relative content of C14:0, C18:2, and C18:3 was enhanced and the relative proportion of C16:0 and C18:1 was decreased, this can be related to the improvement of LA and GLA (Figure 3D), and there were no significant changes in the relative contents of C16:1 and C18:0. Further, the GLA content of Mc-d61 was increased by 61% (from 13 to 21.0%), whereas for Mc-d62 it was only 20% (from 13 to 15.7%).

It has been reported there are two isozymes ∆6-desaturases (Δ61- and Δ62-desaturase) in *Mucor* spp. (Michinaka et al., 2003). Due to ∆6-desaturases being structurally conserved, we identified two ∆6-desaturases sequence in the genome of WJ11 based on homology with some functionally annotated ∆-6 desaturases. The result of the alignment of amino acid sequence (Supplementary Figure 3) revealed that Δ6-desaturase isozymes in WJ11 have a low homology (23.3% at the amino acid level) and their phylogenetic positions are far apart, which was consistent with that there was only 22.8% homology of Δ6-desaturase isozymes in *M. circinelloides* HUT1121 (Michinaka et al., 2003). According to the position of the phylogenetic tree (Supplementary Figure 2), the Δ61-desaturase of WJ11 was close to the Δ61-desaturase of CBS277.49 and HUT1121 and the Δ6-desaturase of *Mortierella. alpina*, indicating that Δ61-desaturase was a fungal type desaturase. While the Δ62-desaturase of WJ11 was rather closer to the borage desaturase.

### The effect of co-overexpression of Δ6- and Δ12-desaturase on GLA production

**Figure 4:**
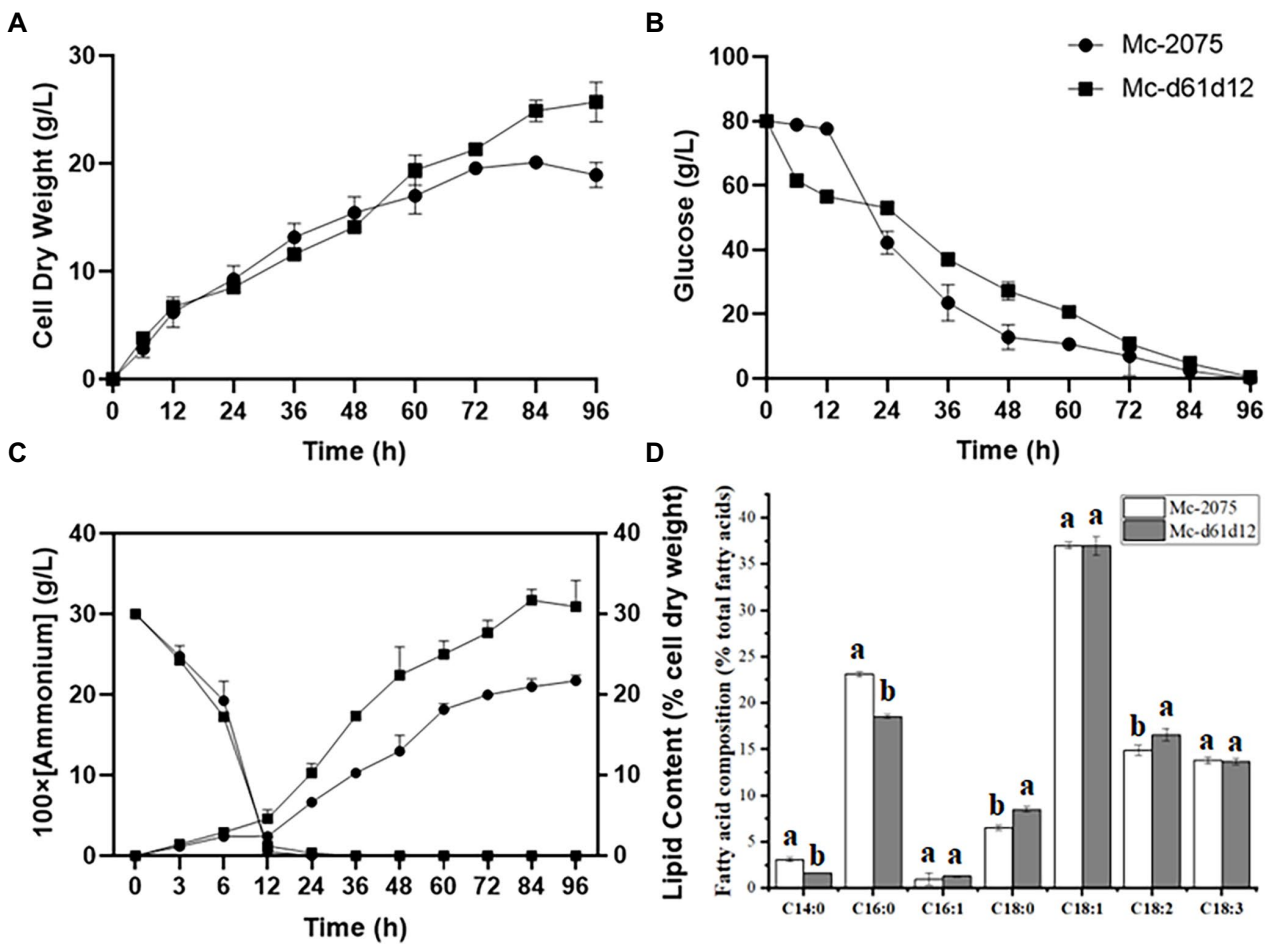
Fermentation parameters of the engineered strains with *DES61* and *DES12* co-overexpression. **(A)** Cell dry weight (CDW), **(B)** Glucose concentration, **(C)** Ammonium concentration and lipid content, **(D)** Fatty acid composition after 72 h fermentation in Mc-2075 (circle and white columns) and Mc-d61d12 (square and gray columns) were measured when cultured in 1 l modified K & R medium for 96 h. The values were mean of three independent replicated experiments. Error bars represent the standard error of mean.

From our results suggested that Δ61-desaturase played a more important role in GLA content, but there were still large amounts of OA accumulation, indicating that the conversion of OA to LA catalyzed by Δ12-desaturase (Wang et al., 2016) may be the rate-limiting step (Zhang et al., 2017). Therefore, we hypothesized that co-overexpressing *DES61* and *DES12* could further increase GLA accumulation through increasing LA as a precursor. As shown in Figure 4, the biomass and lipid content of *DES61* and *DES12* co-overexpressing strains, were higher than that of the control and reached up to 25.8 g/l and 31.7%, respectively (Figures 4A,C). The consumption rate of carbon and nitrogen was the same as the control (Figures 4B,C). As shown in Figure 4D, the LA content was higher than that in the control, while the relative proportion of GLA (12.8%) did not significantly change compared to the control (13%). However, due to the high total fatty acid content and improved biomass, the titer of GLA in Mc-d61d12 reached up to 1.05 g/l as shown in Table 1. As described above, the co-overexpression of *DES61* and *DES12* did increase the GLA yield (Table 1), which was 1.5 folds higher than the control. These results demonstrated that Δ12-desaturase played an important role in LA synthesis and lipid accumulation, which was in accordance with the previous study (Yan et al., 2020) in which overexpression of Δ12-desaturase in *Y. lipolytica* enhanced lipid accumulation.

**Table 1:** The lipid and GLA yields in the control and engineered strains.

Strains	Lipid content (%)	Lipid yield (g/L)	GLA content (%)	GLA yield (g/L)
Mc-2075	22.0 ± 0.06^f^	3.23 ± 0.10^d^	13.0 ± 0.08^de^	0.42 ± 0.01^e^
Mc-d61	24.7 ± 0.19^e^	3.28 ± 0.17^d^	21.0 ± 0.05^a^	0.69 ± 0.04^c^
Mc-d62	27.6 ± 0.06^d^	3.31 ± 0.09^d^	15.7 ± 0.23^c^	0.52 ± 0.11^d^
Mc-d61d12	31.7 ± 0.05^c^	8.20 ± 0.19^a^	12.8 ± 0.16^e^	1.05 ± 0.02^b^
Mc-d61d12d91	38.2 ± 0.20^a^	6.16 ± 0.05^b^	19.8 ± 0.03^b^	1.22 ± 0.01^a^
Mc-d61d12d92	36.0 ± 0.11^b^	5.45 ± 0.17^c^	13.2 ± 0.22^d^	0.72 ± 0.12^c^

Strains were cultured in a 1.5 l fermenter with 1 l K & R medium. The values were means ± SD of three independent replicated experiments. Values with different letters were significantly different to each other.

### The effect of simultaneous overexpression of Δ6-, Δ12-, and Δ9-desaturases on GLA production

**Figure 5:**
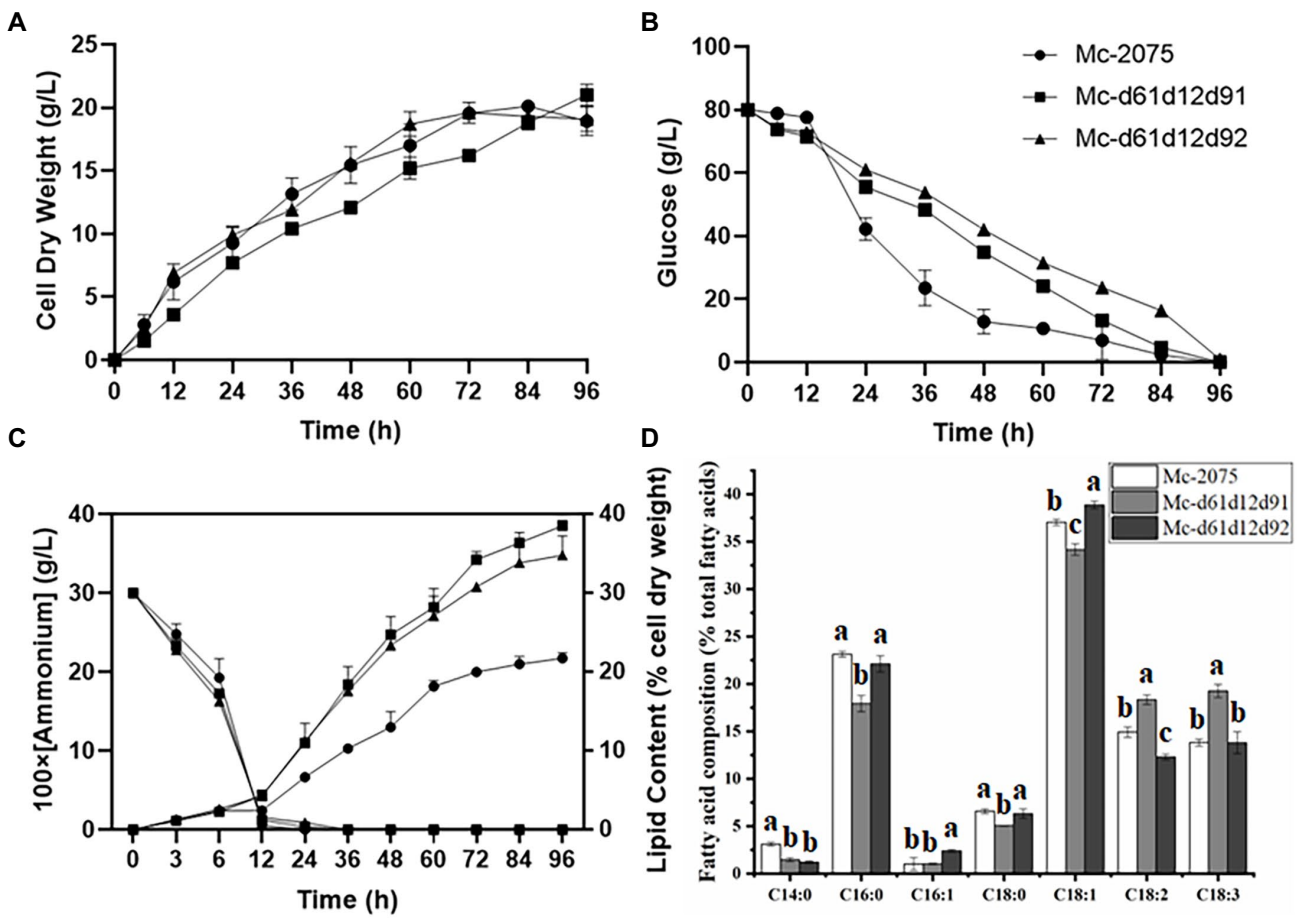
Fermentation parameters of the engineered strains with *DES61*, *DES12*, and *DES91* or *DES92* co-overexpression. **(A)** Cell dry weight (CDW), **(B)** Glucose concentration, **(C)** Ammonium concentration and lipid content, **(D)** Fatty acid composition after 72 h fermentation in Mc-2075 (circle and white columns), Mc-d61d12d91 (square and gray columns) and in Mc-d61d12d92 (triangle and black columns) were measured when cultured in 1 l modified K & R medium for 96 h. The values were mean of three independent replicated experiments. Error bars represent the standard error of mean. Values with different letters were significantly different to each other.

The above experiments demonstrated that co-overexpression of Δ61- and Δ12-desaturases promoted the GLA yield *via* increasing biomass and lipid content. Furthermore, it is well established that Δ9-desaturase also plays a significant role in improving lipid production (Qiao et al., 2015; Tsai et al., 2019). The cell dry weight and the consumption trends of glucose and ammonium of triple overexpression strains (Δ6-, Δ12-, and Δ9-desaturases) were similar to the control strain (Figures 5A–C), which suggested that co-overexpression of Δ6-, Δ12-, and Δ9-desaturases did not affect the growth curve of engineered strains. However, the lipid content of engineered strains was markedly increased, especially in the Mc-d61d12d91, the lipid content was up to 38.2%. And the lipid content in Mc-d61d12d92 was up to 36% (Figure 5C).

The analysis of the fatty acid profile showed that in the Mc-d61d12d91, the content of saturated fatty acids (C14:0, C16:0, and C18:0) and monounsaturated fatty acid (C18:1) decreased significantly, whereas the content of polyunsaturated fatty acids (C18:2 and C18:3) increased significantly compared to the control strain (Figure 5D). However, the content of OA in Mc-d61d12d92 was enhanced sharply while the relative content of GLA was not. Considering that the total fatty acids were improved up to 38.2% and the relative GLA content increased up to 19.8%, the final GLA titer in Mc-d61d12d91 reached 1.22 g/l (Table 1).

### The transcriptional level of the related desaturase genes in engineered strains

**Figure 6:**
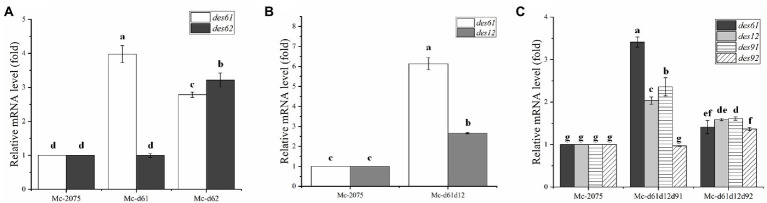
Expression levels of *DES61*, *DES62*, *DES12*, *DES91*, and *DES92* in transformants were determined after 24 h of fermentation by RT-qPCR. **(A)** The expression level of *des61* and *des62* in Mc-2075, Mc-d61, and Mc-d62, **(B)** The expression level of *DES61* and *DES12* in Mc-2075 and Mc-d61d12, **(C)** The expression level of *DES61*, *DES12*, *DES91*, and *DES92* in Mc-2075, Mc-d61d12d91, and Mc-d61d12d92. The values were means of three independent replicated experiments. Error bars represent the standard error of mean. Values with different letters were significantly different to each other.

To analyze the transcriptional level of the relevant desaturase genes by RT-qPCR in the transformants, all the engineered strains were cultured in modified K & R medium for 24 h (rapid lipid accumulation stage). As shown in Figure 6A, the transcriptional level of *DES61* in Mc-d61 was noticeably increased, while the expression level of *DES62* was unchanged compared to the control strain. The transcriptional level of *DES62* was significantly higher in Mc-d62 than that in the control strain, along with a significant increase in the expression of *DES61*. These results indicated that there may be a regulatory relationship between these two Δ6-desaturase isozymes. In Figure 6B, both *DES61* and *DES12* have been overexpressed in Mc-d61d12. And in Mc-d61d12, the expression level of *DES61* was significantly increased in the co-overexpression strains compared to the single overexpression strains, which suggested that the overexpression of *DES12* may have facilitated the expression level of *DES6* through enhancing the precursor LA production. As shown in Figure 6C, the transcriptional levels of *DES61*, *DES12*, *DES91*, and *DES92* were all increased which means both Mc-d61d12d91 and Mc-d61d12d92 accomplished overexpression. Furthermore, the transcriptional level of *DES61*, *DES12*, and *DES91* in Mc-d61d12d91 was the highest, which might contribute to the fatty acid and lipid metabolism.

### Relative expression levels of key genes related to fatty acid desaturation and the NADPH/NADP+ ratio in the engineered strains

Since the biosynthesis of PUFAs is catalyzed by a complex enzyme system consisting of fatty acid desaturases, cytochrome b5, and NADH-dependent cytochrome b5 reductase, meanwhile enzymes providing NADPH for desaturation and elongation must be present. We tried to elucidate the effects of overexpressing desaturase genes on the expression of other key genes involved in the fatty acid synthesis. It is well known that the pentose phosphate pathway (PPP) and malic enzymes play essential roles in providing reducing power NADPH for lipid synthesis in fungus (Zhao et al., 2015). Our previous genome analysis of WJ11 found that there are three genes encoding glucose-6-phosphate dehydrogenase (*g6pdh1*, encoded by scaffold00053.31; *g6pdh2*, encoded by scaffold00034.42; *g6pdh3*, encoded by scaffold00081.31) and two genes encoding 6-phosphogluconate dehydrogenase (*6pgdh1*, encoded by scaffold00113.18; *6pgdh2*, encoded by scaffold00142.5) (Tang et al., 2015b). Malic enzymes can be divided into cytosolic malic enzymes (cMEs) and mitochondrial malate enzymes (mMEs) (Kendrick and Ratledge, 1992a; Vongsangnak et al., 2012). Mitochondrial malate enzymes have been documented to be unrelated to fatty acid synthesis (Li et al., 2007; Zhang et al., 2007). Therefore, the expression level of two genes encoding cytosolic malic enzymes (*cme1*, encoded by scaffold00036.12; *cme2*, encoded by scaffold00049.37) was measured (Vongsangnak et al., 2012). Besides, the gene encoding cytochrome b5 reductase (*cyb5r*, encoded by scaffold00042.2) was analyzed as well.

**Figure 7:**
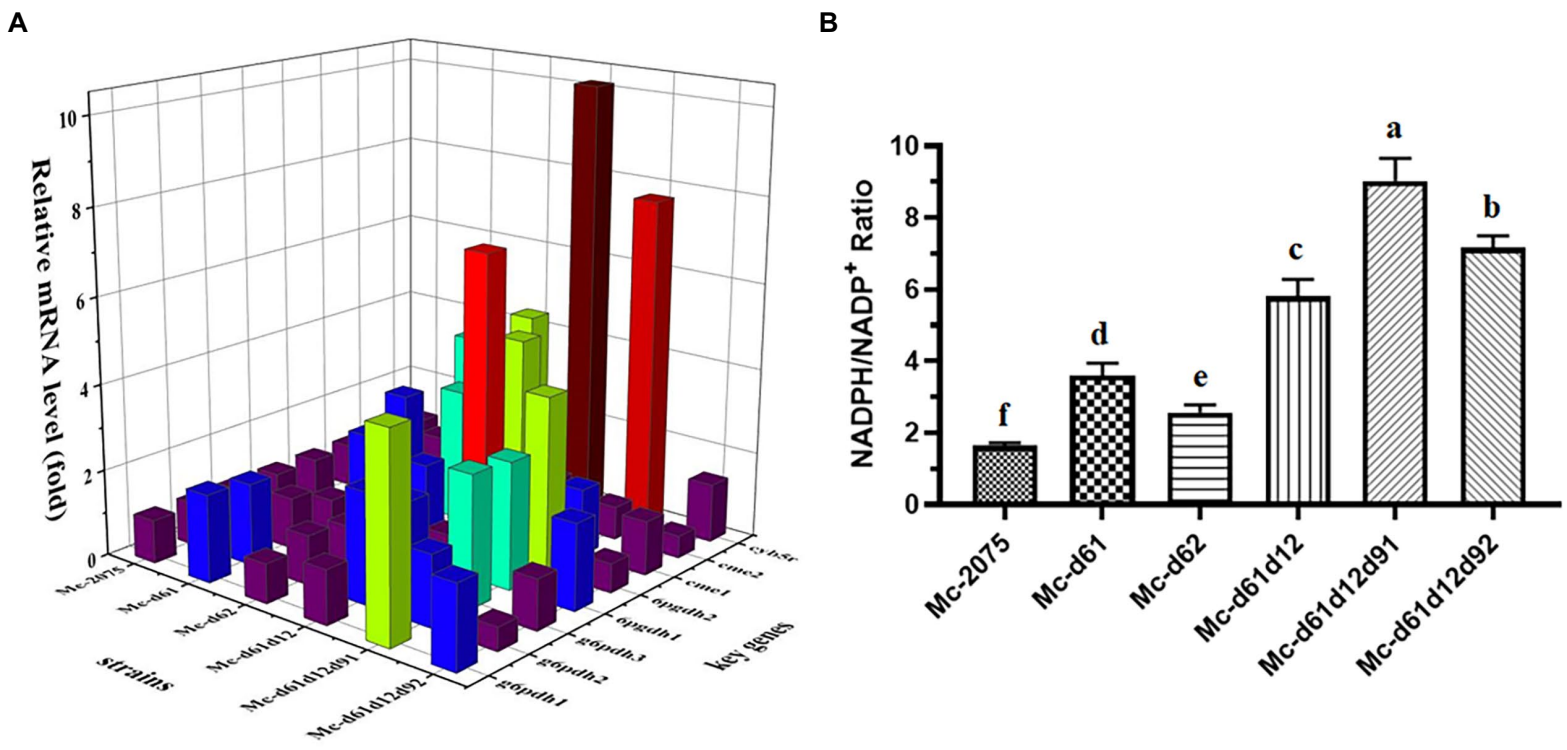
Identification of key factors for GLA synthesis in engineered strains fermented for 24 h. **(A)** Relative expression level of related genes for fatty acid desaturation in all engineered strains. *Cme*, cytosolic malic enzyme; *g6pdh*, glucose-6-phosphate dehydrogenase; *6pgdh*, 6-phosphogluconate dehydrogenase; *cyb5r*, cytochrome b5 reductase. The values were mean of three independent replicated experiments. **(B)** The values of NADPH/NADP^+^ in all engineered strains. The values were mean of three independent replicated experiments. Error bars represent the standard error of mean. Values with different letters were significantly different to each other.

As shown in Figure 7A, many genes encoding key enzymes involved in the desaturation process were up-regulated due to the overexpression of desaturase genes. There were two genes *g6pdh1* and *g6pdh3*, significantly up-regulated in Mc-d61, Mc-d61d12, Mc-d61d12d91, and Mc-d61d12d92, while the transcription level of *g6pdh2* was significantly up-regulated in Mc-d61, Mc-d61d12, and Mc-d61d12d91. For two *6pgdh* genes, *6pgdh1* was only up-regulated in Mc-d61d12d91 and Mc-d61d12d92, while *6pgdh2* was up-regulated in all engineered strains except for Mc-d61d12d92. Moreover, *cme1* was up-regulated in all engineered strains but had no change in Mc-d61d12d92, while the transcription level of *cme2* was only up-regulated in the Δ6-desaturase overexpressing strains. These results suggested that the overexpression of desaturase genes affected the expression of glucose-6-phosphate dehydrogenase, 6-phosphogluconate dehydrogenase, and malic enzymes, thus elevating the levels of lipogenic NADPH in each mutant strain. This was consistent with the NADPH/NADP^+^ ratio measured in mutant strains, which was significantly higher than that of control (Figure 7B). Compared with the control strain, the values were increased by 2.20 folds and 1.57 folds in single overexpression strains (Mc-d61/62), 3.55 folds in dual overexpression strains (Mc-d61d12), 5.52 and 4.37 folds in triple overexpression strains (Mc-d61d12d91/d92). Moreover, the expression level of *cyb5r* was also significantly up-regulated in all engineered strains, especially in Mc-d61d12 and Mc-d61d12d91, which indicated that overexpression desaturase genes promoted the activities of NADH-cytochrome b5 reductase (see Figure 7).

## Discussion

Oleaginous microorganism achieved high lipid accumulation under nitrogen-limiting medium. When nitrogen was depleted, the cells no longer synthesized nucleotides and proteins, thus the remaining carbon source was converted into lipids for storage. *M. circinelloides*, which belongs to filamentous fungus of the class Zygomycetes, is known to be a promising producer of GLA and a potential model organism for elucidating the mechanism of fatty acid desaturation due to its high lipid-producing ability (36% lipid content of cell dry weight) and abilities to utilize a variety of carbon source, including glucose (Ratledge and Wynn, 2002), xylose (Zan et al., 2021), acetate (Du Preez et al., 1995), and cellulose (Zhang et al., 2022). To reveal the GLA-producing pathway in *M. circinelloides*, we overexpressed three key desaturase genes, Δ6-, Δ12-and Δ9-desaturase in this oleaginous fungus, and determined their roles in GLA synthesis of *M. circinelloides*.

We found that single overexpression of two Δ6-desaturase isozymes enhanced the GLA content in different ratio (Figure 3D). From the results, it was observed that Δ61-desaturase could improve GLA content by 61% (from 13 to 21%), while Δ62-desaturase only increased by 20% (from 13 to 15.7%), which implied that Δ61-desaturase played a more significant role in increasing GLA accumulation and it was coincidence with previous study (Zhang et al., 2017). Nevertheless, Δ62-desaturase was phylogenetically close to a plant desaturase, which might explain the low efficiency of Δ62-desaturase in GLA production. Heterologous expression of ∆6-desaturase with high similarity to plant from *M. rouxii* in *S. cerevisiae* provided with exogenous LA, produced only 7.1% GLA of total fatty acids in the mutant (Laoteng et al., 2000). Δ6-desaturase isozymes may differ in response to environmental stress, substrate specificity, and cellular location. For example, when two Δ6-desaturase isozymes of *M. circinelloides* HUT1121 were heterologously overexpressed in *S. cerevisiae*, the transcription level of Mc-D62 was increased at a low temperature (15°C) whereas the expression of Mc-D61 is more than twice of that of Mc-D62 at 28°C (Michinaka et al., 2003). However, Sakuradani *et, al*. heterologous overexpressed two ∆6-desaturase isozymes from *M. alpina* 1S-4 in *Aspergillus oryzae*, respectively, which indicated that one ∆6-desaturase could compensate the other ∆6-desaturase (Sakuradani et al., 1999; Sakuradani and Shimizu, 2003).

In addition, wild-type strain WJ11 was able to accumulate OA (up to 37%), Yang et al. enhanced LA content with OA content decreasing by overexpression ∆12-desaturase, which indicated that ∆12-desaturase was the rate-limiting step (Yang et al., 2022). We predicted that co-overexpression of ∆6- and ∆12-desaturase would further elevate GLA production by increasing the production of precursor LA. But in previous study, the co-expression of ∆6- and ∆12-desaturase was not as effective as the single ∆6-desaturase expression (Zhang et al., 2017). In the present study, the dual overexpression had a higher total fatty acid content (31.7% of CDW) than the single overexpression of *DES61* (24.7% of CDW) and *DES12* (26.8% of CDW) (Yang et al., 2022), but less than the sum of both. The additional introduction of *DES12* in the DES6-overexpressing strain did not increase GLA content (12.8%) compared with the control (13%). Because of the high total fatty acids in Mc-d61d12, the GLA content decreased. In a study by Yan et al., it was found that some desaturases had a function in promoting lipid accumulation by co-overexpressing of ∆12-and ∆9-desaturase, which successfully increased lipid content from 25 to 34% compared to the control (Yan et al., 2020). Multi-gene overexpression increased biomass through enhancing the lipid accumulation, which explained the higher biomass of co-overexpression strains compared to the control. However, the single overexpression strain of ∆6-desaturase was *LeuA* defective, although leucine was added for fermentation, it still affected its growth. In the present study, it was shown that co-overexpression of endogenous ∆6- and ∆12-desaturases increased GLA yield (1.05 g/l) by significantly enhancing the lipid content (from 22 to 31.7%), meanwhile co-overexpression ∆9-desaturase could further increase GLA yield (1.22 g/l). ∆9-desaturase is responsible for introducing the first double bond into a saturated fatty acid and has moderate activity with both C16:0 and C18:0 as substrates. The sequence difference between the two ∆9-desaturase isozymes indicates their difference in evolution and function (Hao et al., 2015). In Mc-d61d12d91, GLA content was significantly increased (from 13 to 19.8%) which indicated that *DES61*, *DES12* and *DES91* could synergistically promote GLA accumulation. In Mc-d61d12d92, although GLA content did not change significantly, *DES92* promoted the conversion ratio of C16:0 to C16:1(from 1 to 2.5%) and C18:0 to C18:1(from 36.1 to 39.2%). The overexpression of a single desaturase gene had a limited effect on GLA synthesis, whereas the co-overexpression of Δ6-and Δ12-desaturase significantly increased the lipid content without altering GLA content. Co-overexpression of three desaturase genes led to a nearly two-fold increase in GLA production through preventing the buildup of intermediates. These results also indicated the role of desaturation pathways in lipid synthesis and suggested that the lipid production can be enhanced by regulating the distribution of fatty acids profile.

To our knowledge, this is the first time to improve GLA yields by simultaneously overexpressing the endogenous Δ6-, Δ12-, and Δ9-desaturase genes. In order to obtain co-overexpression strains of Δ6- and Δ12-desaturases, two expression vectors were constructed and then integrated into the genome of WJ11 by homologous recombination. As for simultaneous overexpression of Δ6-, Δ12-, and Δ9-desaturases, besides single overexpression of Δ61-desaturase, we constructed another vector which was inserted both Δ12- and Δ9-desaturase gene expression cassettes (in the form of “promoter1-gene1-terminator-promoter2-gene2”). Chuang et al. used this tandem gene expression cassettes in Y. lipolytica to achieve GLA production by co-overexpression of Δ6- and Δ12-desaturase, in which the order of the two gene expression cassettes did not affect expression efficiency (Chuang et al., 2010). However, the insertion of a very large DNA structure (~10 kb) and reverse transcription seemed to be a challenge in filamentous fungi. Therefore, this strategy provides a new pathway to express several different genes or multiple copies of one gene.

The overexpression of endogenous Δ6-, Δ12-, and Δ9-desaturase genes increased the lipid content and GLA yields in *M. circinelloides* which were in agreement with the up-regulated expression levels of *DES6*, *DES12*, and *DES9*. Although the transcription level of desaturase genes was slightly different in mutants, which may be due to the addition of other genes that might be responsible for change in expression (Zhang S. et al., 2016). Besides, it has been reported that a high NADPH/NADP^+^ ratio indicates a more reductive cellular environment and may facilitate lipid biosynthesis (Guerra et al., 2013). The supply of NADPH can drive desaturation and elongation reactions, leading to the synthesis of polyunsaturated fatty acids. NADPH can be produced by a few enzymes, of which malate enzymes as well as glucose-6-phosphate dehydrogenase and 6-phosphogluconate dehydrogenase in PPP pathway are the main candidates. The overexpression of desaturases may affect the expression of lipogenic NADPH, thereby increasing the level of GLA. Therefore, the expression levels of relative genes encoding those enzymes were up-regulated differentially in each mutant. Nevertheless, cytochrome b5 reductase is well established as an indispensable enzyme for fatty acid biosynthesis, which uses NADH as the electron donor to catalyze the reduction of cytochrome b5 and then cytochrome b5 transfers electrons to activate desaturase (Zhang Y. et al., 2016). Many desaturases contain cytochrome b5-like N-terminal extension. Classical model holds that cytochrome b5 receives an electron from cytochrome b5 reductase and transfer to cytochrome b5 domain of desaturases before arrived at the active site of desaturase (Certik et al., 1998). In a study by Zhang et al., the disruption of cytochrome b5 reductase caused desaturase dysfunction and led to reduced synthesis of PUFAs (Zhang Y. et al., 2016). In this study, overexpression of key desaturase genes resulted in upregulation of *cyb5r*, suggesting a possible regulatory relationship between them. These results indicate that the fatty acids desaturation process requires a multiple-gene expression system and support the hypothesis that there is a large demand of reducing power and electron donors during the desaturation process in the engineered strains. Collectively, these findings suggested that the upregulation of desaturase gene expression may not be sufficient and multiple enzymes may act synergistically to promote lipid synthesis.

The lipid content of 38.2% (of CDW) and GLA (1.22 g/l) were only obtained in a small fermenter (1 l) and under non-optimized fermentation conditions, which laid the foundation for large-scale production of GLA. Increasing aeration, decreasing the fermentation temperature, and adding nutrients such as malic acid can be used to improve the GLA content. Previously, heterologous expression of Δ15-desaturase and Δ6-elongase enabled the production of stearidonic acid (SDA) and dihomo-gamma-linolenic acid (DGLA) in *M. circinelloides* (Khan et al., 2019a,b). Therefore, further strategies should focus on optimizing fermentation and multi-gene combinations to produce high-value of PUFAs. This study has paved the way for potential industrial production of microbial GLA and provided some new insights into the regulation of PUFA production in filamentous fungi.

## Conclusion

The overexpression of *des61* promoted the relative content of GLA, which was increased by 61.5% than the control. The co-overexpression of *des61* and *des12* improved the GLA yields from 0.42 g/l to 1.05 g/l, which was 1.5 folds higher than the control. Moreover, the simultaneous overexpression of *des61*, *des12*, and *des91* not only enhanced the GLA content (up to 19.8%), but also resulted in a final GLA titer of 1.22 g/l, which was 1.9 folds higher than the control. These results indicated that overexpressing desaturases played an important role in lipid accumulation and fatty acid desaturation. This study provides a new strategy for the development of high-value polyunsaturated fatty acids producing cell factories.

## Data availability statement

The original contributions presented in the study are included in the article/Supplementary material, further inquiries can be directed to the corresponding author.

## Author contributions

XW and JY were involved in the experimental design, manuscript writing, and graphical arrangement. HM, SL, SP, and CW carried out the additional experimental work. FX, WS, AS, and BS reviewed the manuscript. YS proposed the project and reviewed the final manuscript. All authors contributed to the article and approved the submitted version.

## Funding

This work was supported by National Natural Science Foundation of China (grant nos. 31972851 and 32101927), Shandong Postdoctoral Innovation Project (202103031), and Doctor and Postdoctoral Foundation of Shandong University of Technology and Shandong Provincial Key Technology R&D Plan (nos. 2018GNC110039 and 2018GSF121013).

## Conflict of interest

The authors declare that the research was conducted in the absence of any commercial or financial relationships that could be construed as a potential conflict of interest.

## Publisher’s note

All claims expressed in this article are solely those of the authors and do not necessarily represent those of their affiliated organizations, or those of the publisher, the editors and the reviewers. Any product that may be evaluated in this article, or claim that may be made by its manufacturer, is not guaranteed or endorsed by the publisher.

## Supplementary material

The Supplementary material for this article can be found online at: https://www.frontiersin.org/articles/10.3389/fmicb.2022.1078157/full#supplementary-material
