# Supplementary material for: Simultaneous overexpression of ∆6-, ∆12- and ∆9-desaturases enhanced the production of γ-linolenic acid in Mucor circinelloides WJ11
Source: Front Microbiol. 2022 Dec 15;13:1078157. doi: 10.3389/fmicb.2022.1078157 (PMC9797528; doi:10.3389/fmicb.2022.1078157)

**Supplementary** **Table S1**. Primers and their sequences used in this study

| Primers | Sequences (5’-3’) | annotation |
| --- | --- | --- |
| F1 | CGATGGCATGCCCGGGGCGTATGGG  CCCTTATGATATCACAC | Simple cloning of *sodit-a* gene (*Sph I*) |
| R1 | CTGCATACGTACCCGGGGTTACATTA  GCAGGTATTTTTGC | Simple cloning of *sodit-a* gene (*SnaB I*) |
| F2 | CCCGAGCTAGCCTCGAGCCAGATAC  TGTACTTCCACTTTTTAC | Simple cloning of *sodit-a* gene up-stream (*Xba I*) |
| R2 | CGTACTCTAGATTTGTATACAAAGTG  AAAGAAAGATG | Simple cloning of *sodit-a* gene down-stream (*Nhe I*) |
| D61-F | ATGTCTACATCAGGTAAAAAGCGC | Simple cloning of *Des61* gene (*Xho I*) |
| D61-R | TACAATTAAGTAAGAAGGCCTTTTAA | Simple cloning of *Des61* gene (*Xho I*) |
| D62-F | ATGAGCAGCGACGTAGGAGCAACAGC | Simple cloning of *Des62* gene (*Xho I*) |
| D62-R | AGAAATTCAGCAAAAAAATGCTCTAA | Simple cloning of *Des62* gene (*Xho I*) |
| D12-F | ATGATGGCAACCAAGAGAAACGTT | Simple cloning of *Des12* gene (*Xho I*) |
| D12-R | TGTCTTCTTTAAGAACTAATCTATGA | Simple cloning of *Des12* gene (*Xho I*) |
| D91-F | ATGTCAACTACAACGACAACTA | Simple cloning of *Des91* gene (*Xho I*) |
| D91-R | AACCTGAGAAGAACAAGACCAAGTAA | Simple cloning of *Des91* gene (*Xho I*) |
| D92-F | ATGCTCCTTATTACCTGCGCCGAC | Simple cloning of *Des92* gene (*Xho I*) |
| D92-R | CAATAAGCTTTAAGCCTAGAGTTTAA | Simple cloning of *Des92* gene (*Xho I*) |
| CarRP-F | GATAAGCATA AACCAGATCT GC | Amplification of fragment on plasmid |
| CarRP-R | GTATC TGACA TAGTC GAGCT TC | Amplification of fragment on plasmid |
| sodita-F | TTATTTTTATCGTTTGGTGGTACAC  AAACTGC | Amplification of fragment on plasmid |
| sodita-R | GTAACCCCACAGAAATAGAGCCAT  AAGG | Amplification of fragment on plasmid |
| rt-10.52-F | CCTTGTCATGGTGGAAGAACAAGC | RT-qPCR for *Des61* gene located in genome |
| rt-10.52-R | CGGCAATGAAACGGGATAACATGG | RT-qPCR for *Des61* gene located in genome |
| rt-3.2-F | GCAGAGACGCTACAGATGTCTTTC | RT-qPCR for *Des62* gene located in genome |
| rt-3.2-R | CGAATTTCTTGAGCAAAAGCAGCAG | RT-qPCR for *Des62* gene located in genome |
| rt-248.11-F | CAGGTCTTTGTCCCCTCTACTCG | RT-qPCR for *Des12* gene located in genome |
| rt-248.11-R | ATCTTGACCAGAGACATTGGTG | RT-qPCR for *Des12* gene located in genome |
| rt-5.3-F | AAGACATTGTACTGGGCAATCACC | RT-qPCR for *Des91* gene located in genome |
| rt-5.3-R | CACGATGTCCACGAGACCACC | RT-qPCR for *Des91* gene located in genome |
| rt-126.15-F | ACCCTTGAATTCAGATACGGAACTGTG | RT-qPCR for *Des92* gene located in genome |
| rt-126.15-R | TTGTATGTAGTGGCATCAGACATCATGG | RT-qPCR for *Des92* gene located in genome |
| rt-53.31-F | ATTGTTTCACAGAGGAAGAGACTTTCC | RT-qPCR for *g6pd1* gene located in genome |
| rt-53.31-R | TGTTATCAATGTTGGAACGGTTCCAG | RT-qPCR for *g6pd1* gene located in genome |
| rt-34.42-F | TGTCATGCAAAATCATTTGCTGCAG | RT-qPCR for *g6pd2* gene located in genome |
| rt-34.42-R | GACATACTGTCCCAAGAGAGAATCTT  CC | RT-qPCR for *g6pd2* gene located in genome |
| rt-81.31-F | TGCACTCTTCACTGAAAATGAGATTT  ATCG | RT-qPCR for *g6pd3* gene located in genome |
| rt-81.31-R | TTATCAATATAGGTGCGATCCCATGC | RT-qPCR for *g6pd3* gene located in genome |
| rt-113.18-F | GATGGTTCACAACGGTATTGAATACG  GA | RT-qPCR for *6pgd1* gene located in genome |
| rt-113.18-R | CGATCAAGAAAGAATCCAATTCACCC  TTG | RT-qPCR for *6pgd1* gene located in genome |
| rt-142.5-F | GCACAACGGTATTGAGTACGGC | RT-qPCR for *6pgd2* gene located in genome |
| rt-142.5-R | CCCTTATTCCATTCATCAAAGACATC | RT-qPCR for *6pgd2* gene located in genome |
| rt-36.12-F | TGATTCCAAGGGTCTTGTTACTACTA  CACG | RT-qPCR for *cme1* gene located in genome |
| rt-36.12-R | GGACTTGAGTATTAAAGGCACCTGTA  GTAGATG | RT-qPCR for *cme1* gene located in genome |
| rt-49.37-F | TTCCAAGGGTCTTGTGACAATGAACC | RT-qPCR for *cme2* gene located in genome |
| rt-49.37-R | GTGTGTTGAATGCGTTAGGTGTAGGA  GG | RT-qPCR for *cme2* gene located in genome |
| rt-42.2-F | CGCAATGTGCGTCAGTATTCTTCAGG | RT-qPCR for *cyb5r* gene located in genome |
| rt-42.2-R | TTGAACTTCGAGAAGCTTGAGAGAC  AC | RT-qPCR for *cyb5r* gene located in genome |
| actin-F | GATGAAGCCCAATCCAAGA | RT-qPCR for *actin* gene |
| actin-R | TTCTCACGGTTGGACTTGG | RT-qPCR for *actin* gene |

**Supplementary Figure S1**

The construction process of the plasmid pMAT2076.


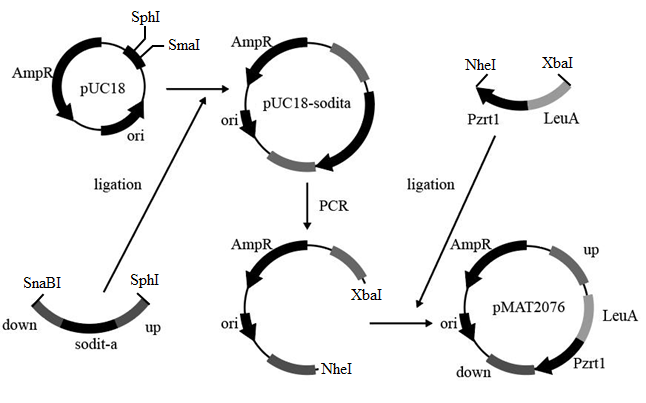


**Supplementary Figure S2**

The maps of empty plasmid pMAT2075, *des61* overexpressing plasmid pCRC124, *des62* overexpressing plasmid pCRC125, empty plasmid pMAT2059, *des12* overexpressing plasmid pCRC129, *des12* and *des91* overexpressing plasmid pCRC150, and *des12* and *des92* overexpressing plasmid pCRC151.


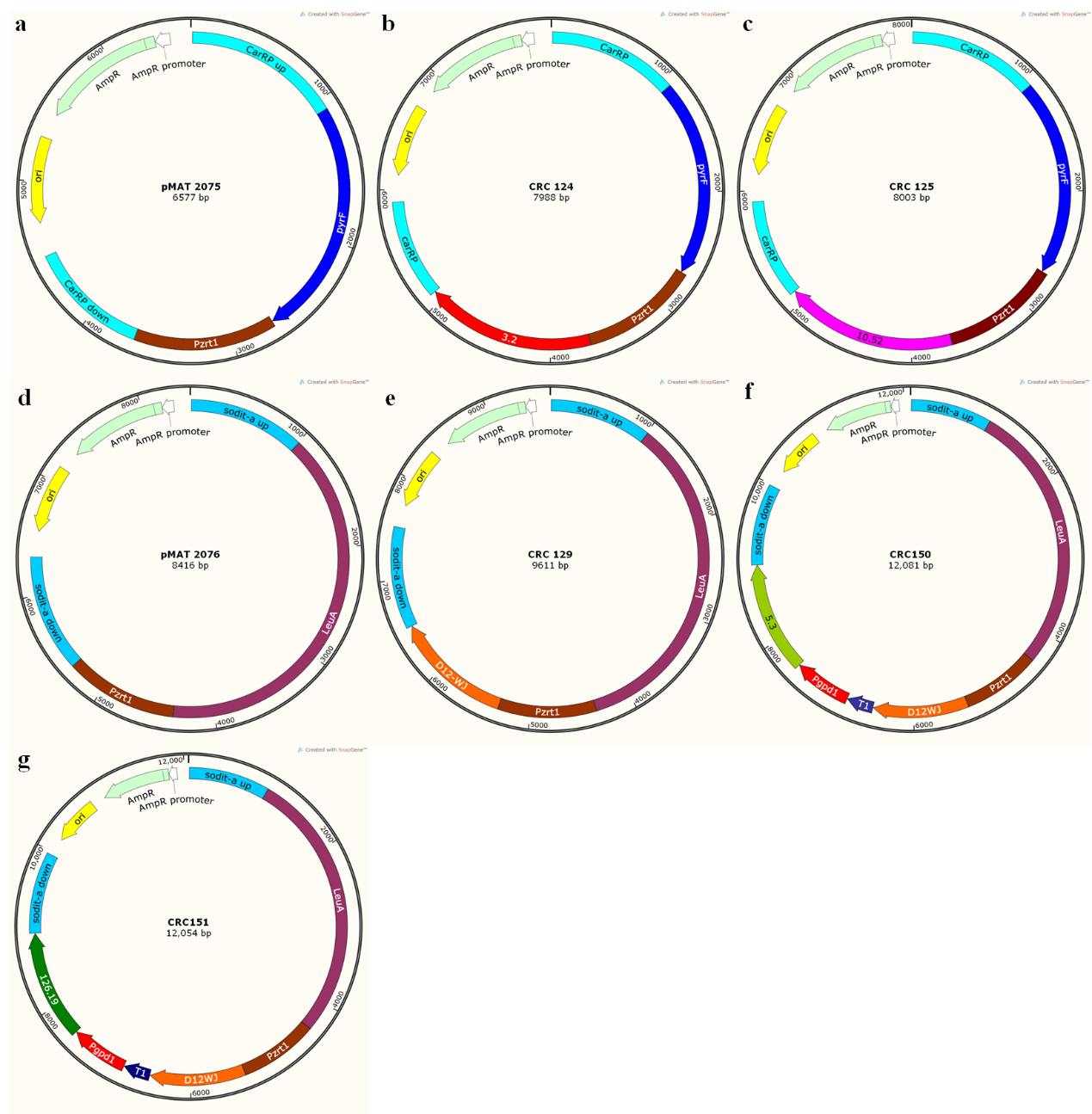


**Supplementary Figure S3**

Alignment of the amino acid sequences and phylogenetic analysis of Δ6-desaturase from *Mucor circinelloides* WJ11 (WJ11-D61 and WJ11-D62), *Mucor lusitanicus* CBS 277.49 (CBS 277.49-D61 JGI accession number ID37214 and CBS 277.49-D62 JGI accession number ID105367), *Mortierella alpina* (DDBJ accession number AB020032), and *Borago officinalis* (DDBJ accession number U79010). Sequence alignment and the construction of phylogenetic tree were done by using CLUSTAL W.


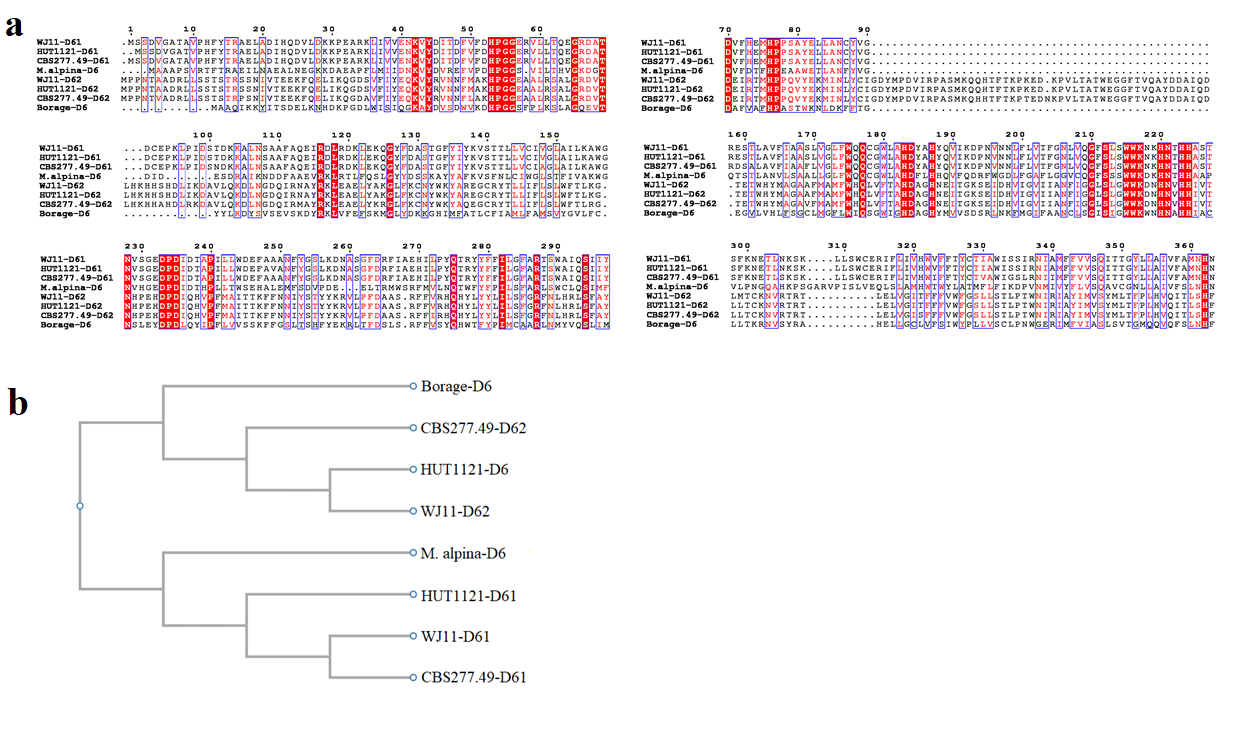

Supplement: Supplementary file 1 [file Table_1.DOCX]
